# Effectiveness and safety of serplulimab plus platinum-based chemotherapy in first-line treatment of extensive-stage small cell lung cancer liver metastases: a retrospective cohort study

**DOI:** 10.3389/fonc.2025.1645692

**Published:** 2025-09-25

**Authors:** Yifei Chen, Wenzhong Su

**Affiliations:** Department of Respiratory Medicine, Shanxi Province Cancer Hospital/Shanxi Hospital Affiliated to Cancer Hospital, Chinese Academy of Medical Sciences/Cancer Hospital Affiliated to Shanxi Medical University, Taiyuan, China

**Keywords:** small cell lung carcinoma, liver neoplasms, immunotherapy, serplulimab, treatment outcome

## Abstract

**Background:**

The role of immunotherapy in patients with extensive-stage small cell lung cancer (ES-SCLC) liver metastases remains controversial. This study aimed to evaluate the effectiveness and safety of serplulimab combined with platinum-based chemotherapy as a first-line treatment for these patients.

**Methods:**

This retrospective cohort study reviewed patients with ES-SCLC liver metastases who received serplulimab plus platinum-based chemotherapy as a first-line treatment. Outcomes included objective response rate (ORR), disease control rate (DCR), progression-free survival (PFS), overall survival (OS), and safety. The associations between prognosis and the depth of remission of primary lung lesions and liver metastases were analyzed.

**Results:**

Among the 30 ES-SCLC patients (median age, 67 years), the ORR was 63.3% (95% CI, 43.9-80.1), and the DCR was 83.3% (95% CI, 65.3-94.4). The median PFS was 5.9 months (95% CI, 4.3-8.1), and the median OS was 9.1 months (95% CI, 6.1-16.2). Patients with a depth of remission of primary lung lesions ≥30% (n=14) had a significantly longer median PFS (8.1 months [95% CI, 6.4-NE] vs. 3.9 months [95% CI, 3.2-6.3], HR:0.28, 95%CI: 0.12-0.65) and OS (16.2 months [95% CI, 9.1-NE] vs. 5.5 months [95% CI, 3.7-13.6], HR: 0.31, 95%CI: 0.12-0.81) than those with a depth of remission <30% (n=16). Patients with a depth of remission of liver metastases ≥30% (n=9) had a significantly extended median OS than those with a depth of remission <30% (n=20) (16.2 months [95% CI, 10.4-NE] vs. 6.3 months [95% CI, 4.4-13.6], HR: 0.21, 95%CI: 0.06-0.73). The most common adverse events were nausea (40.0%), leukopenia (26.7%), and neutropenia (26.7%).

**Conclusion:**

This real-world study demonstrates promising effectiveness and a manageable safety profile for the combination of serplulimab with platinum-based chemotherapy in treating ES-SCLC liver metastases, which suggests that this treatment regimen may provide an attractive option for the first-line management of ES-SCLC liver metastases.

## 1 Introduction

Extensive-stage small cell lung cancer (ES-SCLC) is a highly aggressive subtype of lung cancer, diagnosed in approximately 70% of SCLC patients, and is associated with poor prognosis and a significant disease burden (1). Among patients with ES-SCLC, liver metastasis is particularly common, occurring in around 20-30% of cases (2). Notably, liver metastases tend to develop early in the disease course (3) and are correlated with a significantly worse prognosis compared to other metastatic sites (4). Patients frequently suffer from severe complications due to the extensive nature of liver involvement, including hepatic dysfunction (5). This subset of patients experiences shorter progression-free survival (PFS) and overall survival (OS), poorer performance status, and reduced quality of life (6). Liver metastasis is also often associated with a less favorable response to traditional chemotherapy, further challenging disease control (6). The progression of the disease and resulting comorbidities significantly impact patients’ quality of life, frequently leading to increased symptom burden, higher levels of palliative care needs, and substantial healthcare costs (7, 8).

Current therapeutic strategies for ES-SCLC generally incorporate immunotherapy in combination with platinum-based chemotherapy as the first-line treatment (9, 10). Compared to chemotherapy alone, chemo-immunotherapy demonstrated superior efficacy in prolonging OS in ES-SCLC patients with liver metastasis (11). However, liver metastases have been shown to attenuate the therapeutic benefits. ES-SCLC patients with liver metastases often demonstrate less pronounced clinical improvements from immunotherapy compared to those without liver involvement. It is likely due to the unique immunologic and vascular characteristics of the liver that may hinder effective immune response activation against tumor cells (12).

In recent years, serplulimab, a novel PD-1 inhibitor, has been approved in China for SCLC treatment (13). The ASTRUM-005 clinical trial demonstrated that serplulimab combined with platinum-based chemotherapy improved outcomes for ES-SCLC patients with liver metastases, achieving a median OS of 10.8 months and a median PFS of 6.9 months (14). However, the efficacy of this regimen may vary in real-world settings, where patients often have poorer baseline performance status and greater comorbidities than those in clinical trials. Although guidelines have incorporated serplulimab plus platinum-based chemotherapy for first-line ES-SCLC treatment, there remains a lack of real-world data to assess its safety and effectiveness comprehensively, particularly in patients with liver metastases. This study aims to fill this gap by evaluating the real-world effectiveness and safety of this combination therapy in ES-SCLC patients with liver metastases.

## 2 Methods

### 2.1 Study design and patients

This retrospective cohort study included patients with ES-SCLC and concurrent liver metastases, who underwent first-line treatment with serplulimab in combination with chemotherapy at Shanxi Cancer Hospital from May 2022 to May 2023. Patients were adults aged ≥18 years with histologically or cytologically confirmed ES-SCLC, liver metastases verified by imaging, and at least one measurable lesion per the Response Evaluation Criteria in Solid Tumors (RECIST) version 1.1. Exclusions were patients with composite SCLC or incomplete clinical data (such as pathological diagnosis, treatment details, and outcomes). Ethical approval was granted by the Institutional Review Board of Shanxi Cancer Hospital (Ethics approval number: KY2024162), and the requirement for informed consent was waived due to the study’s retrospective nature.

### 2.2 Data collection

Patient demographic and clinical data were collected using the hospital’s electronic medical record system, including age, sex, weight, smoking history, Eastern Cooperative Oncology Group (ECOG) performance status, pulmonary comorbidities, the sites and number of tumor metastases, and the number of organs involved. The time from diagnosis to the initiation of first-line therapy, as well as the specifics of the treatment regimen and dosage, were also collected. Additionally, the investigators re-evaluated tumor response using patients’ imaging data, including contrast-enhanced computerized tomography or magnetic resonance imaging scans stored in the patient’s medical record system. In cases of discrepancy between the original clinical assessment and the research reevaluation, another independent investigator was consulted for adjudication to determine the final response outcome. The Imaging evaluation was conducted every 6 weeks ± 7 days during the combination therapy phase and was performed based on routine clinical follow-up and physician discretion after completion of combination therapy. Survival data were gathered through outpatient follow-up data and telephone interviews.

### 2.3 Treatment strategy

Patients received serplulimab in combination with platinum-based chemotherapy. Based on clinical indications, patients received either an etoposide and carboplatin (EC) or etoposide and cisplatin (EP) regimen. After completion of initial combination therapy, patients who did not experience disease progression received serplulimab maintenance therapy every three weeks until disease progression, intolerable toxicity.

### 2.4 Outcomes

The outcomes included objective response rate (ORR), disease control rate (DCR), time to progression (TTP), duration of response (DoR), PFS, and OS. These outcomes were evaluated by investigators using the RECIST version 1.1 criteria. ORR was determined by the percentage of patients achieving either a complete response (CR) or a partial response (PR), while DCR was calculated as the proportion of patients exhibiting CR, PR, or stable disease (SD). TTP was defined as the interval from the initiation of treatment to the onset of disease progression, and DoR was the period from the initial CR or PR until progressive disease (PD) or death. OS was measured from the commencement of therapy to death from any cause, and PFS was defined as the duration from treatment initiation to PD or death, whichever occurred first. The depth of remission was defined as the percentage of tumor reduction from baseline for the target lesions.

Adverse events (AEs) were recorded and categorized according to the Common Terminology Criteria for Adverse Events (CTCAE) version 5.0, with specific documentation of immune-related adverse events (irAEs).

### 2.5 Statistical analysis

All statistical analyses were conducted using SPSS software, version 22.0. We evaluated the distribution of continuous data for normality using the Shapiro-Wilk test. Data adhering to normal distribution were expressed as mean ± standard deviation, whereas data with skewed distribution were presented as medians with interquartile ranges (range). Categorical variables were reported as frequencies and percentages. Time-to-event variables, such as survival times, were analyzed using the Kaplan-Meier method, with the generation of Kaplan-Meier curves to illustrate patient prognoses across different subgroups. Comparisons between groups were performed using the log-rank test. Confidence intervals (CI) of 95% for estimated ORR and DCR were computed employing the Clopper-Pearson method.

Subgroup analyses were performed to evaluate the impact of various clinical factors on PFS and OS. Factors analyzed included age group (<65 vs. ≥65), number of metastatic lesions (≤3 vs. >3), and number of liver metastatic lesions (≤3 vs. >3), the presence of bone and adrenal gland metastases, the chemotherapy regimen (EP vs. EC), number of treatment cycles (<4 vs. ≥4), the presence of maintenance treatment (yes vs. no), and depth of remission for neither primary lung lesions or liver metastases (<30% vs. ≥30%, defined based on RECIST v1.1). Univariate Cox regression analyses were conducted to assess the association between various clinical factors and survival outcomes. The hazard ratios (HRs) and 95% CIs were calculated to identify factors significantly associated with PFS and OS. For all statistical tests, a two-sided significance level of α=0.05 was applied.

## 3 Results

### 3.1 Baseline characteristics

This study included 30 patients with ES-SCLC liver metastases who were treated with first-line serplulimab in combination with chemotherapy. The cohort predominantly comprised males (96.7%), with a median age of 67 years (range, 28-76). Among these patients, 66.7% were aged 65 years or older. A family history of tumors was noted in 6.7% of the patients. The majority of patients (90.0%) had an ECOG performance status of 1, with 0 and 2 represented at 6.7% and 3.3%, respectively. A history of respiratory tract disease was documented in 13.3% of the cohort, including chronic obstructive pulmonary disease (3.3%), interstitial lung disease (3.3%), asthma (3.3%), and other respiratory conditions (3.3%). Metastatic distribution involved the lymph nodes in 96.7% of patients, while bone and adrenal metastases were each present in one-third of the cohort (33.3%). Smaller fractions of patients exhibited brain metastases (6.7%) and contralateral lung metastases (6.7%). All patients had liver metastases, with 76.7% having more than three liver metastatic lesions (**Table 1**).

**Table 1:** Baseline characteristics of patients.

Variable	Number (N = 30)
Gender, n (%)	
Male	29 (96.67)
Female	1 (3.33)
Age (years), median (range)	67 (28, 76)
Age (years), n (%)	
<65	10 (33.33)
≥65	20 (66.67)
Family history of tumors, n (%)	
Yes	2 (6.67)
No	28 (93.33)
Smoking, n (%)	
Yes	26 (86.67)
No	4 (13.33)
ECOG PS, n (%)	
0	2 (6.67)
1	27 (90.00)
2	1 (3.33)
History of respiratory tract disease, n (%)	
Yes	4 (13.33)
COPD	1 (3.33)
interstitial lung disease	1 (3.33)
Asthma	1 (3.33)
Other	1 (3.33)
No	26 (86.67)
Clinical stage, n (%)	
IVb	30 (100.00)
Cumulative number of organs, median (range)	4 (3, 6)
Cumulative number of organs, n (%)	
3	11 (36.67)
4	14 (46.67)
5	3 (10.00)
6	2 (6.67)
The number of metastatic lesions, median (range)	4 (2, 5)
The number of metastatic lesions, n (%)	
2	3 (10.00)
3	11 (36.67)
4	13 (43.33)
5	3 (10.00)
Sites of metastases, n (%)	
Liver	30 (100.00)
Lymph node	29 (96.67)
Bone	10 (33.33)
Adrenal gland	10 (33.30)
Brain	2 (6.67)
Contralateral lung	2 (6.67)
The number of liver metastatic lesions, n (%)	
≤3	7 (23.30)
>3	23 (76.70)

ECOG, Eastern Cooperative Oncology Group performance status; COPD, chronic obstructive pulmonary disease.

### 3.2 Treatment condition

A total of 70.0% of patients were treated with EP, while 30.0% received EC. Serplulimab was administered at a median dose of 250 mg (range, 200–300 mg), with 46.7% of patients receiving 200 mg, 6.7% receiving 250 mg, and 46.7% receiving 300 mg. The median number of treatment cycles was 4 (range, 1-6). Maintenance treatment with serplulimab was administered to 33.3% of patients, while 66.7% did not receive maintenance therapy (**Table 2**).

**Table 2:** Treatment pattern.

Treatment	Number (N = 30)
Treatment plan, n (%)	
Serpluimab + EC	9 (30.00)
Serpluimab + EP	21 (70.00)
Serplulimab dose, median (range)	250 (200, 300)
Serplulimab dose, n (%)	
200	14 (46.67)
250	2 (6.67)
300	14 (46.67)
Maintenance treatment, n (%)	
Yes	10 (33.33)
No	20 (66.67)
Treatment cycle, median (range)	4 (1, 6)
Follow-up time, median (range)	17.27 months (2.1-17.27)

EP, etoposide and cisplatin; EC, etoposide and carboplatin.

### 3.3 Effectiveness

The treatment response among the 30 patients is illustrated in **Supplementary Figure 1**. Nineteen patients (63.3%) achieved a PR, six patients (20.0%) had SD, and five patients (16.7%) experienced PD. The ORR was 63.3% (95% CI, 43.9-80.1), and the DCR was 83.3% (95% CI, 65.3-94.4) (**Table 3**).

**Table 3:** Effectiveness.

Outcomes	N=30
Tumor response, n (%)	
Complete response	0
Partial response	19 (63.33)
Stable disease	6 (20.00)
Progressive disease	5 (16.67)
Objective response rate, (95% CI), %	63.33 (43.86-80.07)
Disease control rate, (95% CI), %	83.3 (65.28-94.36)
Median progression-free survival (95% CI), months	5.94 (4.33-8.10)
6-month rate (95% CI), %	50.0 (34.96-71.5)
9-month rate (95% CI), %	15.0 (6.21-36.3)
1-year rate (95% CI), %	NE
Median overall survival (95% CI), months	9.13 (6.13-16.17)
6-month rate (95% CI), %	66.7 (51.8-85.9)
9-month rate (95% CI), %	53.1 (37.9-74.5)
1-year rate (95% CI), %	33.2 (18.6-59.4)

CI, confidence interval; NE, not estimable.

**Figure 1:**
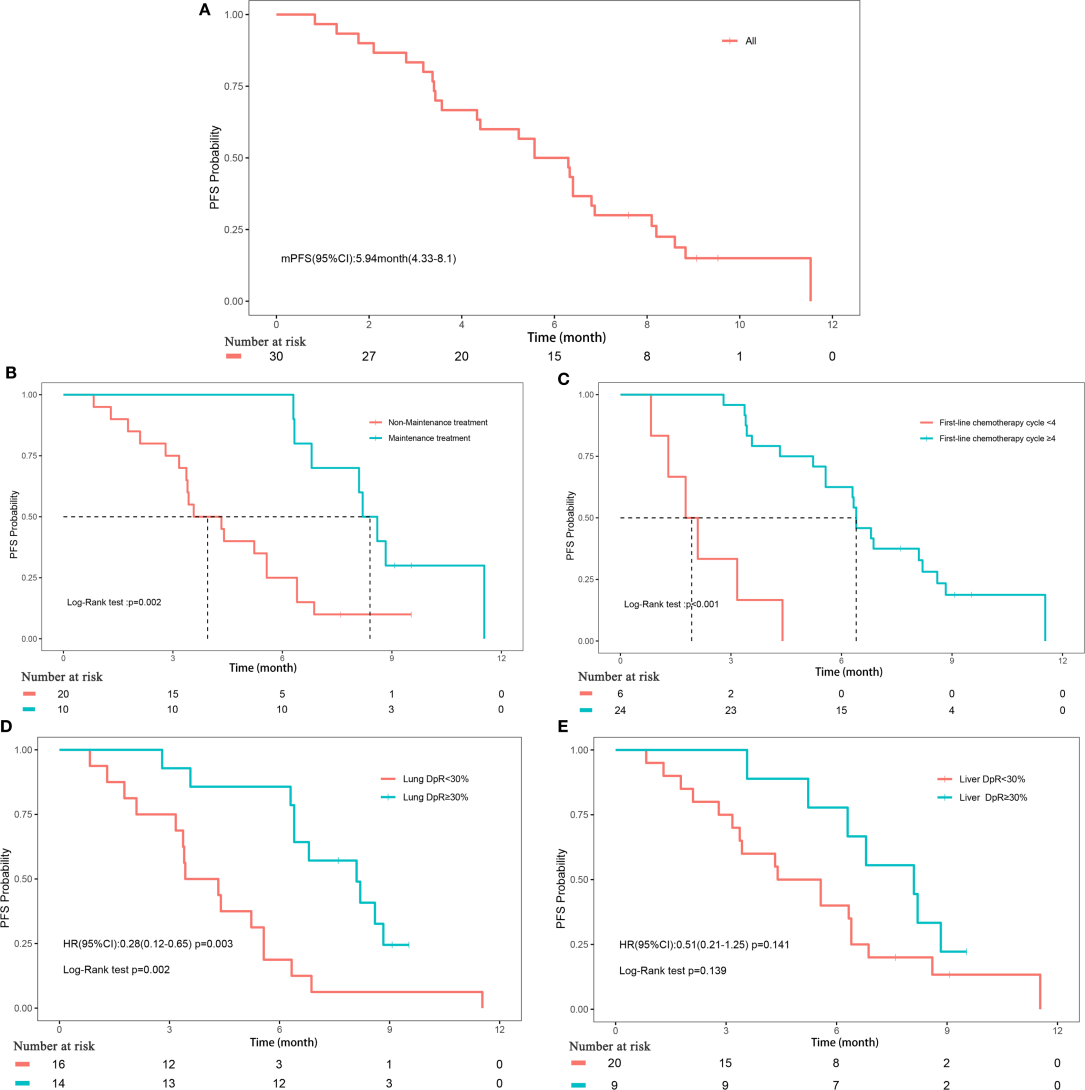
Progression-free survival. **(A)** Progression-free survival of all patients; **(B)** Subgroup analyses of maintenance treatment; **(C)** Subgroup analyses of treatment cycle; **(D)** Subgroup analyses of depth of remission of primary lung lesions; **(E)** Subgroup analyses of depth of remission of liver metastases.

**Figure 2:**
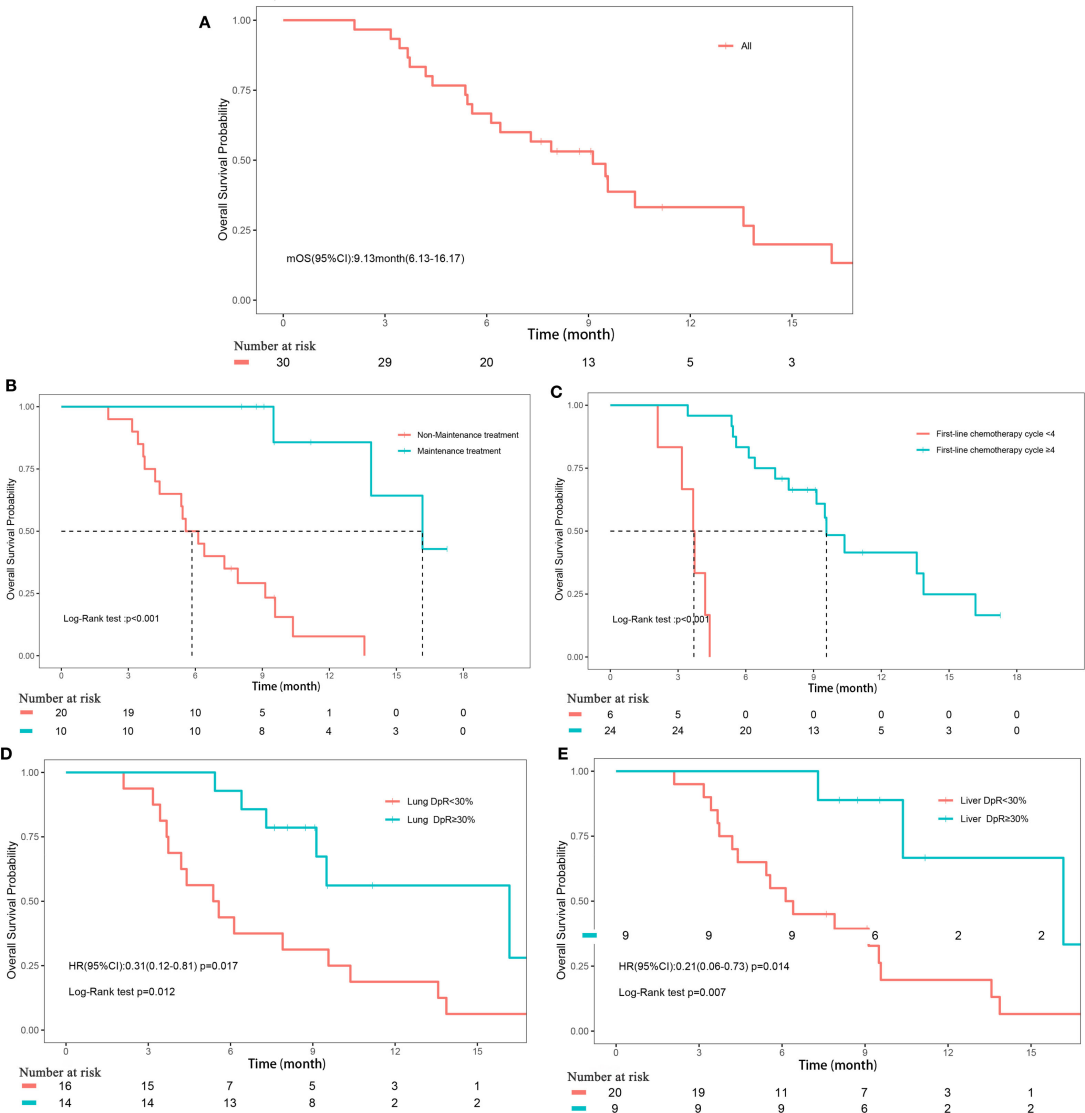
Overall survival. **(A)** Overall survival of all patients; **(B)** Subgroup analyses of maintenance treatment; **(C)** Subgroup analyses of treatment cycle; **(D)** Subgroup analyses of depth of remission of primary lung lesions; **(E)** Subgroup analyses of depth of remission of liver metastases.

The median follow-up duration for the cohort was 17.3 months (range, 2.1-17.3 months). PFS events were observed in 26 patients (86.67%), with a median PFS of 5.9 months (95% CI, 4.3-8.1). OS events occurred in 21 patients (70%), with a median OS of 9.1 months (95% CI, 6.1-16.2) (**Figures 1A**, **2A**). The median DoR was 4.8 months (95% CI, 3.4-not estimable [NE]), and the median TTP was 7.5 months (95% CI, 6.3-NE). Given that two patient deaths were attributable to COVID-19, a sensitivity analysis of OS was conducted by excluding those two patients. The results of sensitivity analysis (median OS: 9.13 months; 95% CI: 6.3-NE) were consistent with those from the primary analysis of the entire cohort, suggesting the OS results of our study are robust (**Supplementary Figure 2**).

The depth of remission varied notably between primary lung lesions and liver metastases among the study cohort. For primary lung lesions, the mean depth of remission was -39.2% (± 39.67%), with a median reduction of -28.7% (range, -100% to 44.44%) (**Supplementary Figure 3A**). In liver metastases, the mean depth of remission was -34.21% (± 50.12%), with a median reduction of -44.12% (range, -100% to 163.16%) (**Supplementary Figure 3B**). Subgroup analyses revealed that maintenance treatment with serplulimab, a higher number of treatment cycles (≥4), and a depth of remission of primary lung lesions ≥30% were all significantly associated with improved PFS (all P<0.05; **Figures 1B–E**, **Supplementary Figure 4**). For OS, significant factors included maintenance treatment with serplulimab, treatment cycle count (≥4), depth of remission of primary lung lesions ≥30%, and depth of remission of liver metastases ≥30% (all P<0.05; **Figures 2B–D**, **Supplementary Figure 5**).

Univariate Cox regression analysis further supported these findings, demonstrating that treatment cycles ≥4 (HR, 0.08; 95% CI, 0.03-0.27; P<0.001), maintenance treatment with serplulimab (HR, 0.25; 95% CI, 0.1-0.64; P = 0.004), and depth of remission of primary lung lesions ≥30% (HR, 0.28; 95% CI, 0.12-0.65; P = 0.003) were significantly associated with longer PFS. For OS, treatment cycles ≥4 (HR, 0.02; 95% CI, 0-0.17; P<0.001), maintenance treatment with serplulimab (HR, 0.05; 95% CI, 0.01-0.36; P = 0.003), depth of remission of primary lung lesions ≥30% (HR, 0.31; 95% CI, 0.12-0.81; P = 0.017), and depth of remission of liver metastases ≥30% (HR, 0.25; 95% CI, 0.1-0.64; P = 0.003) were all significantly predictive of longer survival times (**Table 4**).

**Table 4:** Univariate Cox regression analysis of survival.

Variable	Progression-free survival		Overall survival	
	HR (95%CI)	P value	HR (95%CI)	P value
Age				
<65	Reference		Reference	
≥65	1.23 (0.54-2.8)	0.616	2.16 (0.78-5.96)	0.137
Cumulative number of organs	1 (0.62-1.62)	0.986	1.13 (0.66-1.92)	0.658
The number of metastatic lesions	0.74 (0.46-1.19)	0.214	0.93 (0.53-1.64)	0.800
The number of metastatic lesions				
≤3	Reference		Reference	
>3	0.54 (0.24-1.23)	0.144	1.03 (0.43-2.43)	0.954
The number of liver metastatic lesions				
≤3	Reference		Reference	
>3	1.91 (0.7-5.18)	0.205	2.14 (0.71-6.5)	0.178
Bone metastases				
No	Reference		Reference	
Yes	0.9 (0.4-2.05)	0.808	0.59 (0.23-1.54)	0.281
Adrenal gland metastases				
No	Reference		Reference	
Yes	0.5 (0.2-1.2)	0.120	1.02 (0.4-2.57)	0.972
Treatment plan				
Serpluimab +EP	Reference		Reference	
Serpluimab +EC	1.09 (0.46-2.54)	0.849	0.63 (0.21-1.87)	0.403
Treatment cycle				
<4	Reference		Reference	
≥4	0.08 (0.03-0.27)	<0.001	0.02 (0-0.17)	<0.001
Maintenance treatment				
No	Reference		Reference	
Yes	0.25 (0.1-0.64)	0.004	0.05 (0.01-0.36)	0.003
Depth of remission of primary lung lesions				
<30%	Reference		Reference	
≥30%	0.28 (0.12-0.65)	0.003	0.31 (0.12-0.81)	0.017
Depth of remission of liver metastases				
<30%	Reference		Reference	
≥30%	0.46 (0.19-1.09)	0.077	0.25 (0.1-0.64)	0.003

30% represents a 30% reduction from baseline.

### 3.4 Safety

As summarized in **Table 5**, AEs of any grade occurred in 19 patients (63.3%). The most common AE reported included nausea (40.0%), followed by leukopenia (26.7%) and neutropenia (26.7%). Grade 3 or higher AEs occurred in 2 patients (6.7%), including leukopenia, neutropenia, thrombocytopenia, and pneumonia. Eight patients (26.7%) reported irAEs. Among these, hypothyroidism and elevated aspartate aminotransferase were each reported in 2 patients (6.7%). Other irAEs, such as nausea, vomiting, pneumonia, primary adrenal insufficiency, elevated total bilirubin, and diarrhea, occurred in 1 patient (3.3%) each. During treatment, a total of 17 patients (56.7%) were infected with COVID-19. Among these, 3 patients experienced treatment delays due to COVID-19 infection (by 10, 30, and 14 days, respectively), 2 patients had to discontinue their scheduled chemotherapy because of COVID-19, and two patients died as a result of COVID-19.

**Table 5:** Adverse events.

Events, n (%)	All (N = 30)		
	Any grade	Grade ≥3	irAE
Any AE	19 (63.3)	2 (6.7)	8 (26.7)
Nausea	12 (40.0)	0	1 (3.3)
Leukopenia	8 (26.7)	1 (3.3)	0
Neutropenia	8 (26.7)	1 (3.3)	0
Vomiting	7 (23.3)	0	1 (3.3)
Thrombocytopenia	5 (16.7)	1 (3.3)	0
Pneumonia	3 (10.0)	1 (3.3)	3 (10.0)
Elevated ASL	2 (6.7)	0	2 (6.7)
Hypothyroidism	2 (6.7)	0	2 (6.7)
Anemia	2 (6.7)	0	0
Elevated ALT	2 (6.7)	0	0
Hyperglycemia	2 (6.7)	0	0
Primary adrenal insufficiency	1 (3.3)	0	1 (3.3)
Elevated total bilirubin	1 (3.3)	0	1 (3.3)
Diarrhea	1 (3.3)	0	1 (3.3)
Elevated creatinine	1 (3.3)	0	0

AE, adverse event; irAE, immune-related adverse event; ASL, aspartate aminotransferase; ALT, alanine aminotransferase.

## 4 Discussion

This study substantiates the clinical use of serplulimab combined with platinum-based chemotherapy in treating ES-SCLC liver metastases, demonstrating an ORR of 63.3% and a DCR of 83.3%. The median PFS was 5.9 months, and the median OS was 9.1 months. Subgroup analyses indicated that the depth of remission is a critical factor influencing survival outcomes. Notably, patients achieving a depth of remission of ≥30% in primary lung lesions had significantly longer PFS and OS, while a depth of remission of ≥30% in liver metastases was also associated with prolonged OS. Additionally, the manageable safety profile of this combination supports its feasibility for broad clinical application in ES-SCLC liver metastases.

The advent of PD-1/PD-L1 ICIs has significantly altered the therapeutic landscape for SCLC, challenging the long-standing reliance on chemotherapy as the primary treatment modality (15, 16). PD-1/PD-L1 inhibitors enhance T cell responses and improve the immune system’s ability to target cancer cells by blocking the PD-1/PD-L1 pathway (17). However, this immunological enhancement may have a limited impact in patients presenting with liver metastases, as liver metastases are often characterized by a lower infiltration of cytotoxic T lymphocytes compared to metastases located in the lung, bone, or brain (18). A meta-analysis synthesizing data from four randomized controlled trials highlighted a modest discrepancy in the efficacy of ICIs, revealing that SCLC patients with liver metastases experienced lesser OS benefits from ICI therapy compared to patients without such metastases (HR, 1.22; 95% CI, 1.01-1.46; P = 0.036) (12). This was further supported by a meta-analysis of 14 real-world studies, which identified the presence of liver metastases as an independent adverse prognostic factor for OS in SCLC patients undergoing ICI therapy (P<0.0001) (12). Moreover, outcomes from the CASPIAN study underscored these observations, showing that the addition of durvalumab to a standard EP chemotherapy regimen did not significantly enhance OS in patients with liver metastases compared to those without (HR = 0.87, 95% CI 0.66-1.16) (19). Similar findings were reported in the CAPSTONE-1 study of adebrelimab plus EC, where the OS HR for patients with liver metastases was 0.92 (95% CI 0.55-1.31), suggesting no substantial survival advantage (20). These collective results from various studies illustrate a nuanced landscape where, despite the revolutionary potential of PD-L1 inhibitors (10), their benefits in ES-SCLC patients with liver metastases are inconsistent and limited.

The clinical benefit of serplulimab for SCLC with liver metastases may not be consistent with other PD-1 inhibitors, such as pembrolizumab (21). Serplulimab and pembrolizumab exhibited similar performance *in vitro* and *in vivo* studies, but serplulimab demonstrated consistently superior anti-tumor activity compared to pembrolizumab upon co-administration with anti-TIGIT or anti-LAG3 inhibitors (22). Serplulimab robustly induces PD-1 receptor endocytosis while fostering weaker PD-1-CD28 cis interactions, leading to sustained and robust T cell activation. Therefore, the serplulimab combination effectively reduces tumor microenvironment Treg cell populations, augments effector and memory T cell populations, and more potently modulates genes associated with diverse facets of the immune system, surpassing the effects of the pembrolizumab combination (22). These findings suggest that serplulimab may offer improved efficacy in ES-SCLC liver metastases, highlighting its potential for enhanced therapeutic response in this challenging patient subset.

The ASTRUM-005 study, which assessed serplulimab as a first-line therapy for ES-SCLC, has significantly contributed to the evolving landscape of treatment options for patients with liver metastases. Notably, the subgroup analysis focusing on patients with concomitant liver metastases revealed that serplulimab offers considerable OS benefits, with an HR of 0.58 (95% CI 0.40-0.84), aligning closely with outcomes observed in patients without liver metastases (14, 23). This finding positions serplulimab as a potentially preferred immunotherapy option for treating ES-SCLC patients with liver metastases. Despite these promising clinical trial results, a noticeable gap remains in real-world evidence regarding the effectiveness of immune combination therapies in ES-SCLC liver metastases. The current study, which reported an OS of 9.1 months, highlights this gap, especially considering the poorer ECOG performance scores and the universal presence of metastatic disease among patients.

In contrast to the ASTRUM-005 trial, which studied a diverse group of ES-SCLC patients, including brain and liver metastases, our study population focused on ES-SCLC patients with liver metastases. Patients in this real-world study were older and had poorer ECOG scores. These disparities highlight a patient population with a more advanced disease state and a generally poorer health status, factors that typically predict a diminished response to treatment (24). Moreover, our study was conducted amidst the unprecedented challenges posed by the COVID-19 pandemic. The pandemic’s pervasive impact on healthcare systems worldwide led to logistical hurdles for many of our patients, resulting in treatment delays for 3 patients, discontinuation for 2 patients, and COVID-19-related deaths for 2 patients among those who contracted the virus (n=17, 56.7%). Such disruptions could have potentially skewed our results, given the critical importance of uninterrupted treatment in oncology, particularly for patients with ES-SCLC. Therefore, it is plausible that the therapeutic effectiveness observed in our study may have been underestimated due to these adverse effects of the pandemic. Nevertheless, the fact that our study still managed to secure a substantial antitumor response and a DoR not far off from those observed in a controlled clinical trial setting underscores the potential robustness of the treatment regimen utilized. This comparison not only reaffirms the effectiveness of the therapeutic approach employed in the ASTRUM-005 but also suggests that similar outcomes can be anticipated in real-world settings, even among populations with generally poorer prognostic factors.

Depth of remission has been extensively studied in non-small cell lung cancer (NSCLC) as a predictor of survival outcomes (25, 26), but to date, no comparable research has been conducted for SCLC. In this study, we uniquely evaluated the depth of remission separately for primary lesions and liver metastases, a method that diverges from the single, unified approach typically used in NSCLC studies. Our findings demonstrate that the depth of remission of primary lesions is significantly associated with both PFS and OS, suggesting that the response of primary tumors to immunotherapy reflects the overall systemic response and can serve as a predictor of long-term prognosis. Additionally, depth of remission of liver metastases was correlated with OS, with a similar trend observed for PFS, indicating that depth of remission of liver metastases may also have predictive value for treatment effectiveness. These findings imply that the response mechanisms of primary lesions and liver metastases to serplulimab are aligned, further highlighting the potential of depth of remission as a meaningful marker in SCLC.

In our study, AEs of any grade were reported in 63.3% of patients, predominantly nausea, leukopenia, and neutropenia. The occurrence of grade 3 or higher AEs was limited to 6.7% of patients. Notably, irAEs were observed in 26.7% of participants, which is considerably lower than the rates reported in pivotal trials such as IMpower133, where 39.9% of patients experienced irAEs, primarily rash and hypothyroidism (9). Comparatively, the CASPIAN study reported higher incidences of severe neutropenia and anemia, with irAEs in up to 36% of patients, emphasizing more frequent thyroid-related irAEs (19). Similarly, in the ASTRUM-005 trial, 33.2% of patients experienced significant treatment-related adverse events, such as decreased neutrophil and white blood cell counts (23). The CAPSTONE-1 (20) and KEYNOTE-604 (20) studies also reported substantial hematological AEs and irAEs, including a notable incidence of hypothyroidism. Our study’s relatively low incidence of irAEs, combined with a manageable range of other AEs, underscores the favorable safety profile of the treatment regimen employed, especially when considering its application in patients with typically poor prognoses and advanced disease stages. However, it is important to consider that the incidence of AEs might be underestimated due to the retrospective nature of our study.

Our study has several limitations. A significant constraint is the relatively small sample size, which limits the generalizability of our findings. Additionally, the absence of a control group restricts our ability to draw more definitive causal inferences regarding the effectiveness and safety of the treatment regimen. The retrospective design of our study also introduces potential biases, which could affect the robustness of our conclusions. Notably, while prolonged survival was associated with both the receipt of maintenance therapy and completion of more than four treatment cycles, these associations should be interpreted in the context of inherent immortal-time bias. Patients who remained progression-free and survived longer had a greater opportunity to receive subsequent therapies, indicating that extended treatment exposure is intrinsically linked to longer pre-existing survival. The results require validation using more robust methods, such as time-dependent covariate modeling or landmark analysis in future studies. Furthermore, the follow-up period in our study may not have been sufficient to fully assess long-term outcomes and late-emerging adverse events. These limitations underscore the need for larger, prospective, randomized controlled trials to more accurately evaluate the benefits and risks of this therapeutic approach in a more diverse patient population.

Our study provides valuable real-world clinical evidence demonstrating promising effectiveness and a manageable safety profile for the combination of serplulimab with platinum-based chemotherapy in treating ES-SCLC liver metastases. The remission depth of primary lung lesions and liver metastases hinted at prognosis. The findings demonstrate that this combination can offer a durable antitumor response and a favorable safety profile, highlighting its potential as a viable treatment option for ES-SCLC liver metastases.

## Data Availability

The original contributions presented in the study are included in the article/**Supplementary Material**. Further inquiries can be directed to the corresponding author.

## References

[1] Bray F Laversanne M Sung H Ferlay J Siegel RL Soerjomataram I. Global cancer statistics 2022: GLOBOCAN estimates of incidence and mortality worldwide for 36 cancers in 185 countries. CA Cancer J Clin. (2024) 74:229–63. doi: 10.3322/caac.21834, PMID: 38572751

[2] Wang Q Gümüş ZH Colarossi C Memeo L Wang X Kong CY. SCLC: epidemiology, risk factors, genetic susceptibility, molecular pathology, screening, and early detection. J Thorac Oncol. (2023) 18:31–46. doi: 10.1016/j.jtho.2022.10.002, PMID: 36243387 PMC10797993

[3] Sung H Ferlay J Siegel RL Laversanne M Soerjomataram I Jemal A. Global cancer statistics 2020: GLOBOCAN estimates of incidence and mortality worldwide for 36 cancers in 185 countries. CA Cancer J Clin. (2021) 71:209–49. doi: 10.3322/caac.21660, PMID: 33538338

[4] Li D Xu X Liu J Liang D Shi J Li S. Small cell lung cancer (SCLC) incidence and trends vary by gender, geography, age, and subcategory based on population and hospital cancer registries in Hebei, China, (2008-2017). Thorac Cancer. (2020) 11:2087–93. doi: 10.1111/1759-7714.13412, PMID: 32589361 PMC7396395

[5] Wang WZ Shulman A Amann JM Carbone DP Tsichlis PN. Small cell lung cancer: Subtypes and therapeutic implications. Semin Cancer Biol. (2022) 86:543–54. doi: 10.1016/j.semcancer.2022.04.001, PMID: 35398266

[6] Nakazawa K Kurishima K Tamura T Kagohashi K Ishikawa H Satoh H. Specific organ metastases and survival in small cell lung cancer. Oncol Lett. (2012) 4:617–20. doi: 10.3892/ol.2012.792, PMID: 23205072 PMC3506697

[7] Ganti AKP Loo BW Bassetti M Blakely C Chiang A D’Amico TA. Small cell lung cancer, version 2.2022, NCCN clinical practice guidelines in oncology. J Natl Compr Canc Netw. (2021) 19:1441–64. doi: 10.6004/jnccn.2021.0058, PMID: 34902832 PMC10203822

[8] Oncology Society of Chinese Medical Association, Chinese Medical Association Publishing House. Chinese Medical Association guideline for clinical diagnosis and treatment of lung cancer, (2023 edition). Zhonghua Yi Xue Za Zhi. (2023) 103:2037–74. doi: 10.3760/cma.j.cn112137-20230510-00767, PMID: 37455124

[9] Horn L Mansfield AS Szczesna A Havel L Krzakowski M Hochmair MJ. First-line atezolizumab plus chemotherapy in extensive-stage small-cell lung cancer. N Engl J Med. (2018) 379:2220–9. doi: 10.1056/NEJMoa1809064, PMID: 30280641

[10] Liu SV Reck M Mansfield AS Mok T Scherpereel A Reinmuth N. Updated overall survival and PD-L1 subgroup analysis of patients with extensive-stage small-cell lung cancer treated with atezolizumab, carboplatin, and etoposide (IMpower133). J Clin Oncol. (2021) 39:619–30. doi: 10.1200/jco.20.01055, PMID: 33439693 PMC8078320

[11] Zhang SL Yu J Tian Y Zhang JH Sun L Huang LT. The optimal first-line therapy for extensive-stage small-cell lung cancer based on liver metastasis status: A network meta-analysis and systematic review. Cancer Med. (2024) 13:e70256. doi: 10.1002/cam4.70256, PMID: 39358989 PMC11447196

[12] Xia H Zhang W Zhang Y Shang X Liu Y Wang X. Liver metastases and the efficacy of immune checkpoint inhibitors in advanced lung cancer: A systematic review and meta-analysis. Front Oncol. (2022) 12:978069. doi: 10.3389/fonc.2022.978069, PMID: 36330494 PMC9623244

[13] Lee A. Serplulimab: first approval. Drugs. (2022) 82:1137–41. doi: 10.1007/s40265-022-01740-0, PMID: 35796953

[14] Cheng Y Zhang S Han L Wu L Chen J Zhao P. First-line serplulimab plus chemotherapy in extensive-stage small-cell lung cancer: Updated results and biomarker analysis from the ASTRUM-005 randomized clinical trial. Cancer Commun (Lond). (2025) 45:990–1009. doi: 10.1002/cac2.70032, PMID: 40440184 PMC12365545

[15] Facchinetti F Di Maio M Tiseo M. Adding PD-1/PD-L1 inhibitors to chemotherapy for the first-line treatment of extensive stage small cell lung cancer (SCLC): A meta-analysis of randomized trials. Cancers (Basel). (2020) 12(9):2645. doi: 10.3390/cancers12092645, PMID: 32947924 PMC7565587

[16] Lee JC Green MD Huppert LA Chow C Pierce RH Daud AI. The liver-immunity nexus and cancer immunotherapy. Clin Cancer Res. (2022) 28:5–12. doi: 10.1158/1078-0432.Ccr-21-1193, PMID: 34285059 PMC8897983

[17] Shiravand Y Khodadadi F Kashani SMA Hosseini-Fard SR Hosseini S Sadeghirad H. Immune checkpoint inhibitors in cancer therapy. Curr Oncol. (2022) 29:3044–60. doi: 10.3390/curroncol29050247, PMID: 35621637 PMC9139602

[18] García-Mulero S Alonso MH Pardo J Santos C Sanjuan X Salazar R. Lung metastases share common immune features regardless of primary tumor origin. J Immunother Cancer. (2020) 8:e000491. doi: 10.1136/jitc-2019-000491, PMID: 32591432 PMC7319789

[19] Goldman JW Dvorkin M Chen Y Reinmuth N Hotta K Trukhin D. Durvalumab, with or without tremelimumab, plus platinum-etoposide versus platinum-etoposide alone in first-line treatment of extensive-stage small-cell lung cancer (CASPIAN): updated results from a randomised, controlled, open-label, phase 3 trial. Lancet Oncol. (2021) 22:51–65. doi: 10.1016/s1470-2045(20)30539-8, PMID: 33285097

[20] Wang J Zhou C Yao W Wang Q Min X Chen G. Adebrelimab or placebo plus carboplatin and etoposide as first-line treatment for extensive-stage small-cell lung cancer (CAPSTONE-1): a multicentre, randomised, double-blind, placebo-controlled, phase 3 trial. Lancet Oncol. (2022) 23:739–47. doi: 10.1016/s1470-2045(22)00224-8, PMID: 35576956

[21] Rudin CM Awad MM Navarro A Gottfried M Peters S Csőszi T. Pembrolizumab or placebo plus etoposide and platinum as first-line therapy for extensive-stage small-cell lung cancer: randomized, double-blind, phase III KEYNOTE-604 study. J Clin Oncol. (2020) 38:2369–79. doi: 10.1200/jco.20.00793, PMID: 32468956 PMC7474472

[22] Zhang Y Wei R Song G Yang X Zhang M Liu W. Insights into the mechanisms of serplulimab: a distinctive anti-PD-1 monoclonal antibody, in combination with a TIGIT or LAG3 inhibitor in preclinical tumor immunotherapy studies. MAbs. (2024) 16:2419838. doi: 10.1080/19420862.2024.2419838, PMID: 39497266 PMC11540081

[23] Cheng Y Han L Wu L Chen J Sun H Wen G. Effect of first-line serplulimab vs placebo added to chemotherapy on survival in patients with extensive-stage small cell lung cancer: the ASTRUM-005 randomized clinical trial. JAMA. (2022) 328:1223–32. doi: 10.1001/jama.2022.16464, PMID: 36166026 PMC9516323

[24] Dall’Olio FG Maggio I Massucci M Mollica V Fragomeno B Ardizzoni A. ECOG performance status >/=2 as a prognostic factor in patients with advanced non small cell lung cancer treated with immune checkpoint inhibitors-A systematic review and meta-analysis of real world data. Lung Cancer. (2020) 145:95–104. doi: 10.1016/j.lungcan.2020.04.027, PMID: 32417680

[25] Tas F Ciftci R Kilic L Karabulut S. Age is a prognostic factor affecting survival in lung cancer patients. Oncol Lett. (2013) 6:1507–13. doi: 10.3892/ol.2013.1566, PMID: 24179550 PMC3813578

[26] Hagmann R Zippelius A Rothschild SI. Validation of pretreatment prognostic factors and prognostic staging systems for small cell lung cancer in a real-world data set. Cancers (Basel). (2022) 14(11):2625. doi: 10.3390/cancers14112625, PMID: 35681605 PMC9179878

