# Supplementary material for: Effectiveness and safety of serplulimab plus platinum-based chemotherapy in first-line treatment of extensive-stage small cell lung cancer liver metastases: a retrospective cohort study
Source: Front Oncol. 2025 Sep 25;15:1645692. doi: 10.3389/fonc.2025.1645692 (PMC12507603; doi:10.3389/fonc.2025.1645692)
Supplement: Supplementary file 1 [file DataSheet1.docx]

Supplementary Material

**Supplementary Figures**

**
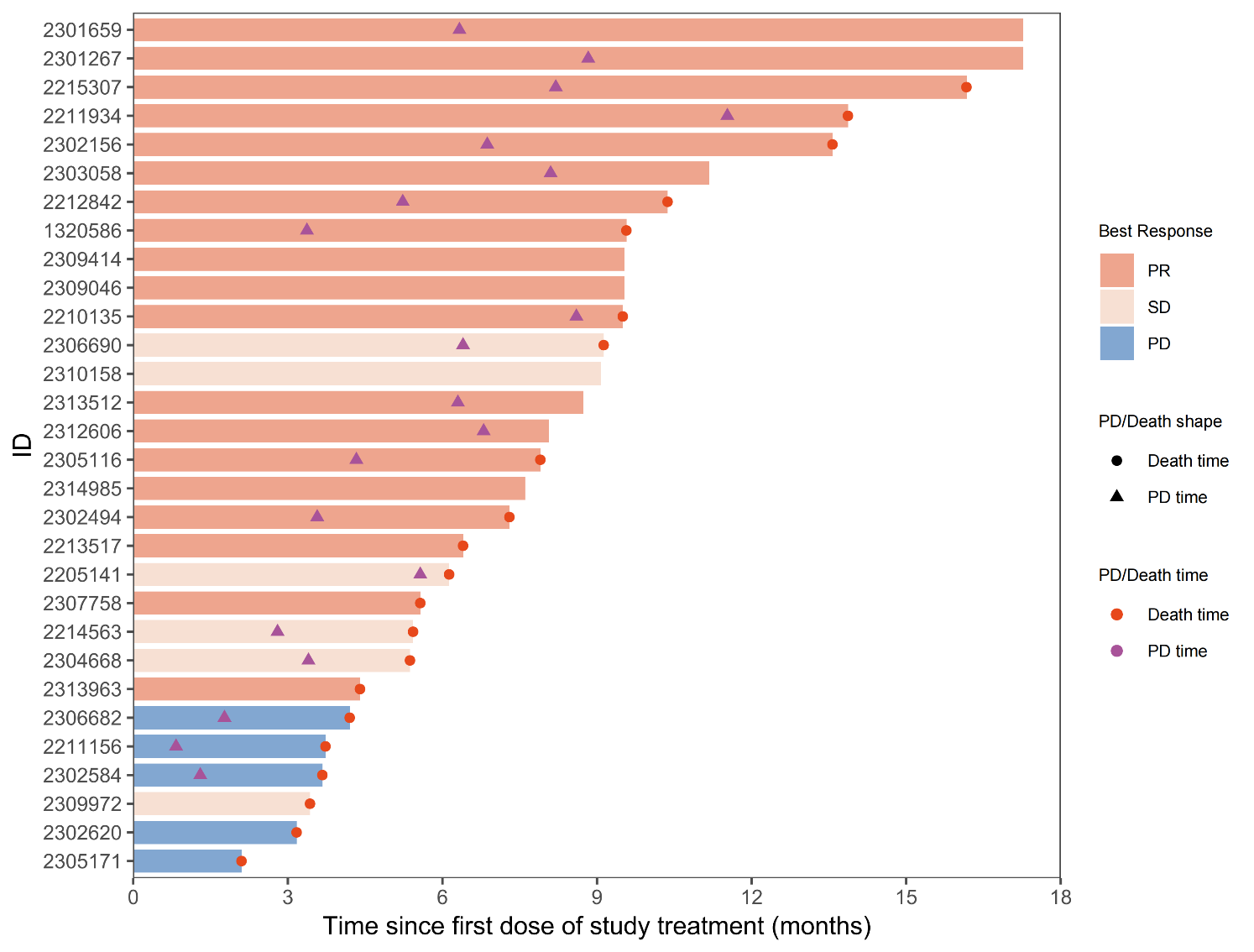
**

**Supplementary Figure 1.** Treatment exposure and duration of response of patients. PR, partial response; SD, stable disease; PD, progressive disease.

**
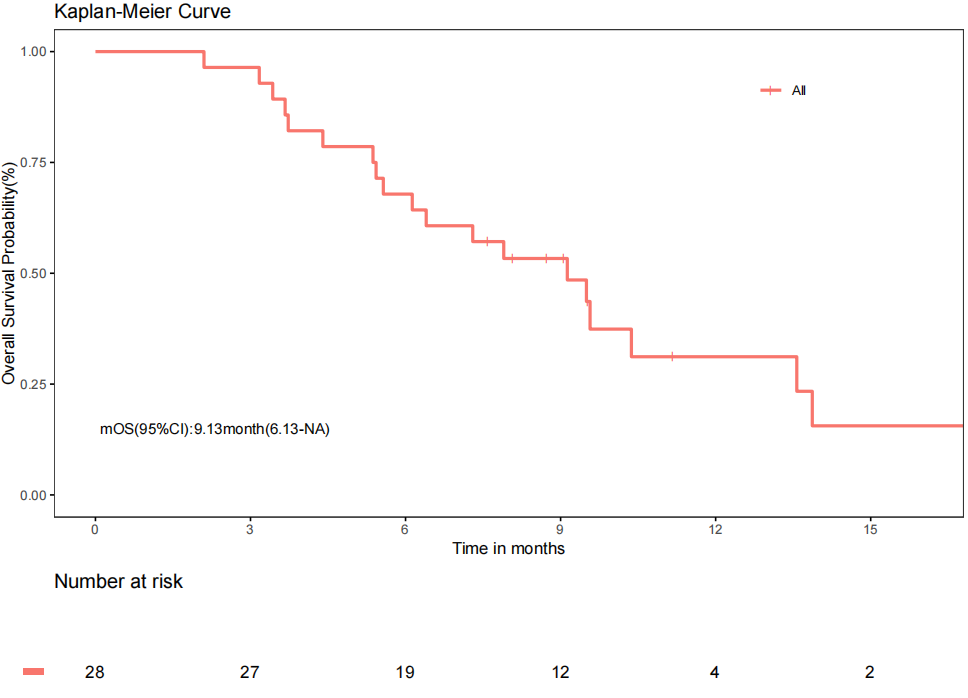
**

**Supplementary Figure 2.** Sensitivity analysis of overall survival by excluding patients who died from COVID-19.

**
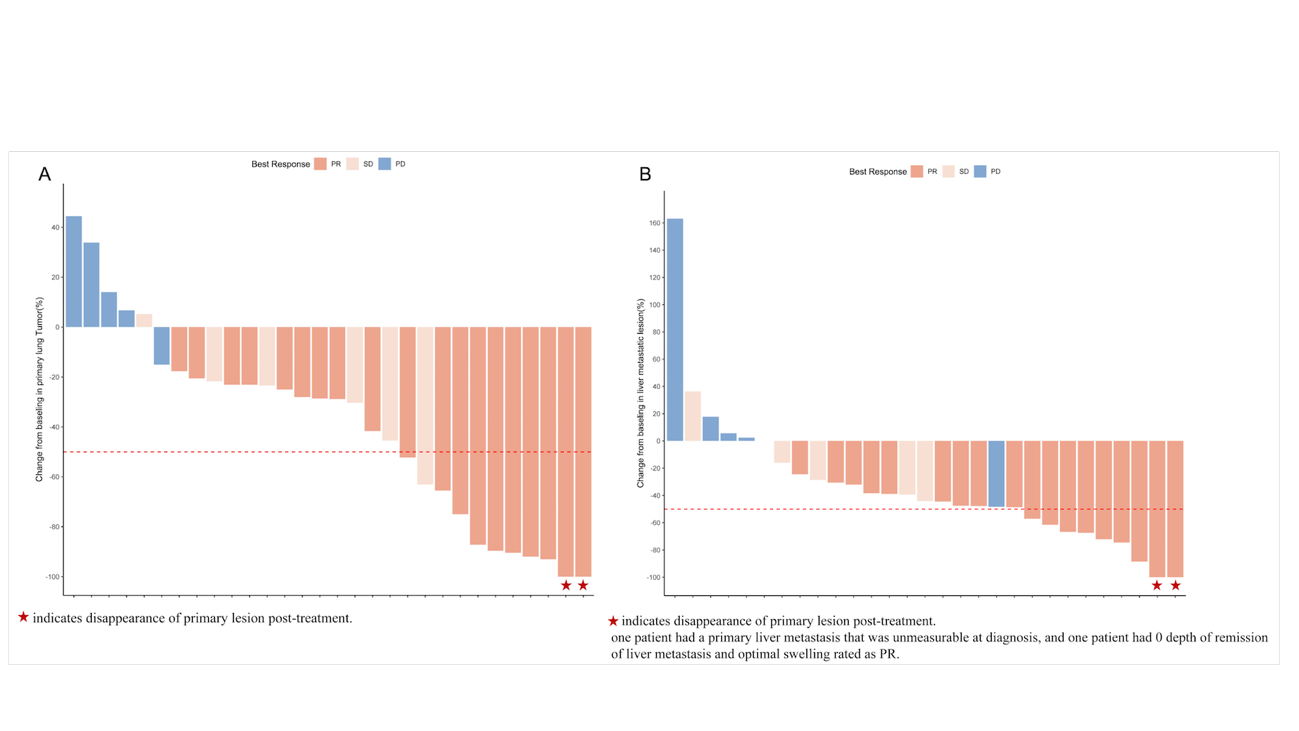
**

**Supplementary Figure 3.** Depth of remission of (A) primary lung lesions; (B) liver metastases.

**
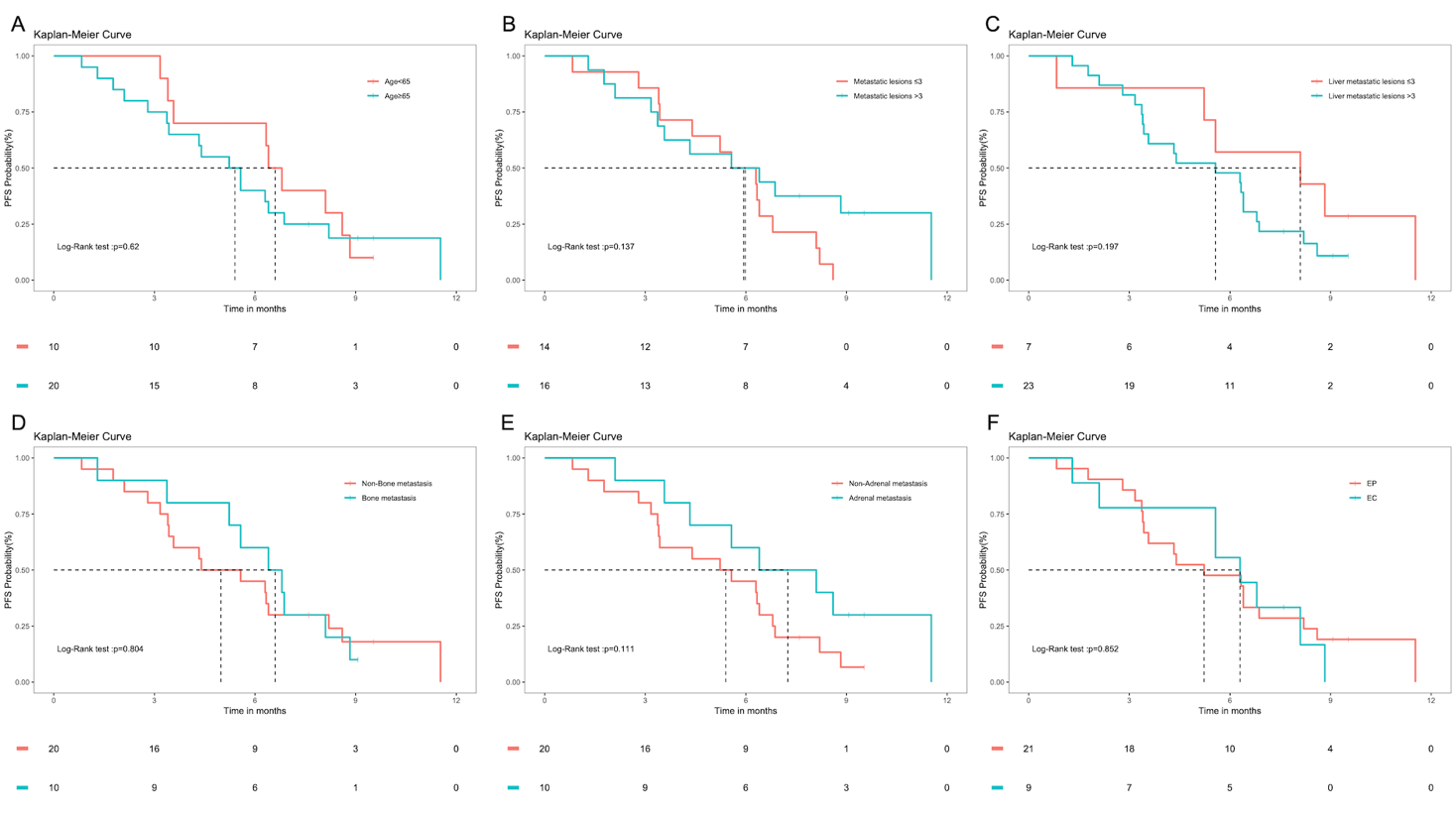
**

**Supplementary Figure 4.** Subgroup analyses of progression-free survival according to (A) age; (B) number of metastatic lesions; (C) number of liver metastatic lesions; (D) presence of bone metastases; (E) presence of adrenal gland metastases; (F) treatment plan.

**
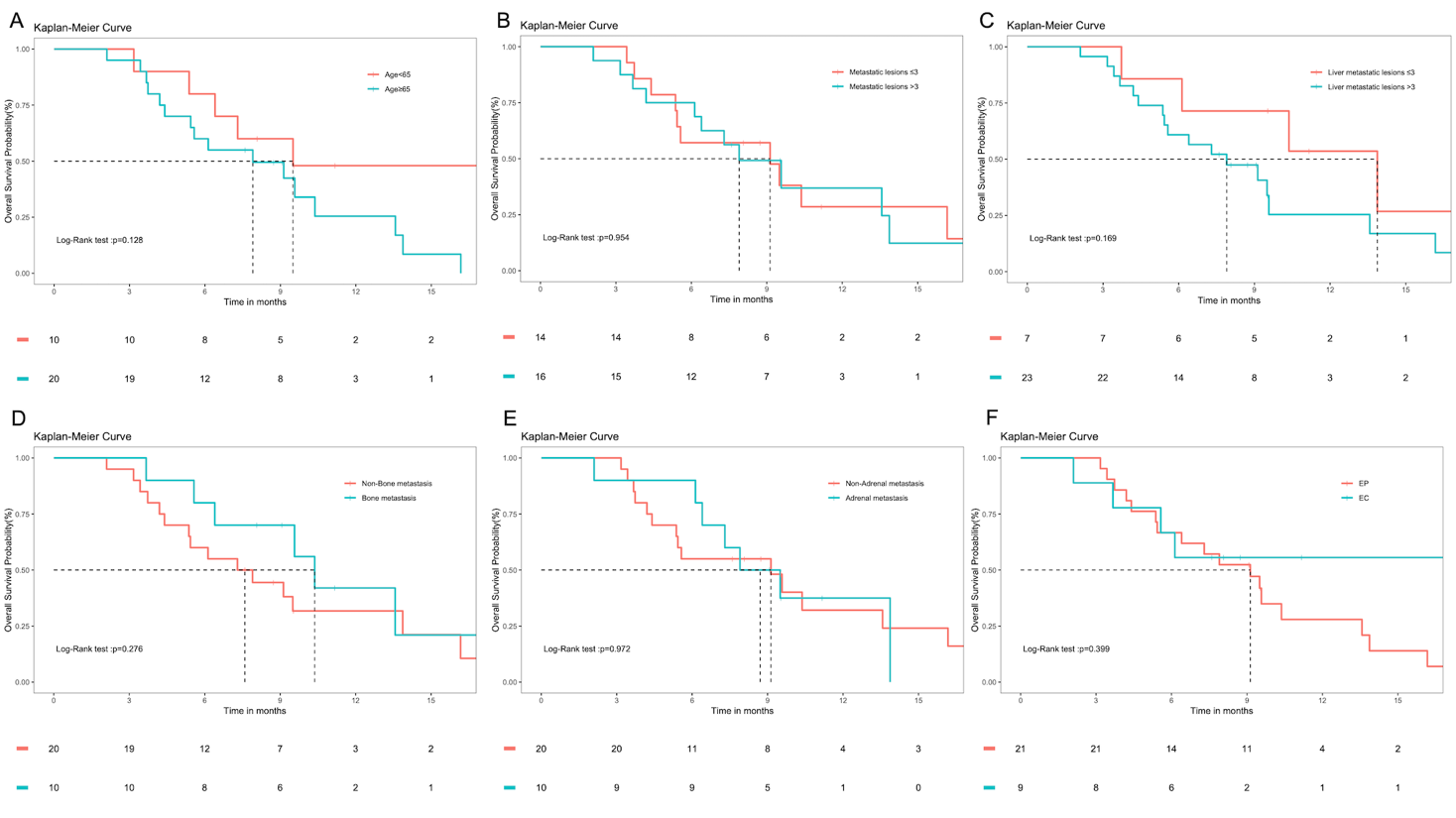
Supplementary Figure 5.** Subgroup analyses of overall survival according to (A) age; (B) number of metastatic lesions; (C) number of liver metastatic lesions; (D) presence of bone metastases; (E) presence of adrenal gland metastases; (F) treatment plan.
